# Effect of Cryopreservation on the Ex Vitro Establishment of Olive Plants Regenerated via Somatic Embryogenesis

**DOI:** 10.3390/plants10020396

**Published:** 2021-02-19

**Authors:** Fatiha Bradaï, Carolina Sánchez-Romero

**Affiliations:** Departamento de Botánica y Fisiología Vegetal, Universidad de Málaga, Campus de Teatinos s/n, 29071 Málaga, Spain; fatihar19@gmail.com

**Keywords:** embryogenic culture, ex vitro acclimatization, in vitro multiplication, in vitro rooting, *Olea europaea*, regeneration potential, somatic embryo

## Abstract

Cryopreservation is considered the best technique for the safe, long-term conservation of embryogenic cultures. However, before integrating it into a somatic embryogenesis system, the influence of cryopreservation on the final production of plants should be investigated. The objective of this investigation was to evaluate the effect of cryopreservation on the regeneration performance of olive embryogenic cultures as well as on the quality of the plants obtained and their response to ex vitro establishment. In order to analyze the influence of the genotype, all the investigations were carried out in two genetically distinct embryogenic lines. The results obtained revealed no variation in the regeneration potential or the quality of the regenerated plants due to cryopreservation. The subsequent multiplication, rooting, and acclimatization steps were not influenced by cryopreservation either, although a significant genotype × cryopreservation interaction was found for shoot length during the multiplication step. The genotype played an important role, determining the quality of the regenerated plants and some aspects of the multiplication and rooting phases. This investigation revealed that the droplet-vitrification procedure optimized for the cryopreservation of olive somatic embryos can be efficiently used for the long-term conservation of olive embryogenic lines.

## 1. Introduction

Olive (*Olea europaea* L.) is one of the most characteristic species around the Mediterranean basin. The value of its products, especially olive oil, makes the olive one of the most economically important fruit tree crops in this part of the world, with a significant influence in the economy of some regions.

The recent expansion of olive outside of its traditional area of cultivation [1] along with the employment of new cultural practices and the demand for improved market quality have greatly increased the demand for improved cultivars [2,3]. The olive breeding programs currently underway are focused on reducing the unproductive period by shortening the juvenile phase and limiting alternate bearing, modifying tree architecture in order to adapt it to intensive cultivation and mechanical harvesting, increasing oil production by increasing fruit number, size, and oil content, and improving oil quality in terms of fatty acid composition, phenol content, and organoleptic characteristics. Promotion of self-fertility and resistance to some biotic and abiotic stresses are also important objectives of olive improvement programs [2,4].

The traditional breeding strategies present substantial limitations to olive improvement, such as long juvenile phase (10–15 years) and reproductive self-incompatibility [5]. Nevertheless, olive genetic improvement can be largely facilitated by the combination of traditional and modern technologies, which have reached an advanced stage of development in recent years [6].

Somatic embryogenesis is a powerful in vitro technique that can be used as a complement in olive breeding by both conventional and unconventional means. Apart from allowing mass clonal propagation of selected genotypes, somatic embryogenesis is the regeneration technique mostly used in woody plants [7] and, therefore, it allows the application of biotechnological tools in these species.

Cryopreservation, i.e., storage at –196 °C, the temperature of liquid nitrogen (LN), is considered the best method for the long-term conservation of plant genetic resources [8], including embryogenic cultures. Cryopreservation is less laborious and costly than maintaining embryogenic cultures by repetitive subcultures [9] and diminishes the risk of contamination and loss of cultures by human error [10]. Furthermore, it avoids the detrimental effects of long-term subculture, such as loss of embryogenic competence or occurrence of somaclonal variation [11].

Somatic embryogenesis and cryopreservation have become significant components of advanced breeding strategies [12]. The combined use of both techniques provides a powerful tool to considerably improve the ability to select superior genotypes in tree species [13]. Thus, cryopreservation allows the safe storage of embryogenic lines while the best genotypes are selected through long-term field trials [14]. Moreover, it permits the storage of a high number of genotypes, preserving their juvenile characteristics until the results of progeny testing become available [15].

In olive, somatic embryogenesis has been repeatedly applied [16], and cryopreservation of somatic embryos has been successfully accomplished by using the droplet-vitrification method on aluminum foil strips [17]. Although the recovery rates obtained after rewarming allow the secure long-term storage of olive embryogenic cultures [18], the effect of cryopreservation on their regeneration potential and the final production of plants is unknown.

The aim of the present investigation was to determine the effect of cryopreservation on the regeneration performance of olive embryogenic cultures as well as on the quality of the regenerated plantlets, evaluating their behavior in the subsequent steps required for ex vitro plant establishment, i.e., shoot multiplication, rooting, and acclimatization. Given the influence of the genotype on embryogenic cultures behavior [19], the investigation was carried out in two independent embryogenic lines.

## 2. Results

### 2.1. Effect of Cryopreservation on the Regeneration Potential

**Figure 1:**
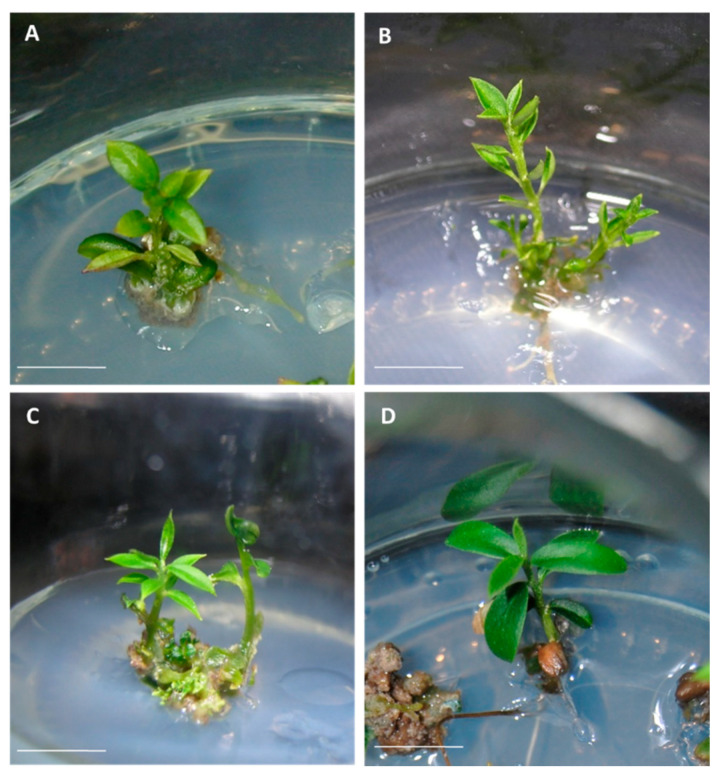
Plantlets regenerated from (**A**,**C**) control and (**B**,**D**) cryopreserved embryogenic cultures of the lines T1 (**A**,**B**) and P5 (**C**,**D**). Bar = 1 cm.

Plantlets obtained from the germination of olive somatic embryos presented a good appearance, independently of the embryogenic line and their origin, from control or cryopreserved cultures (Figure 1).

**Figure 2:**
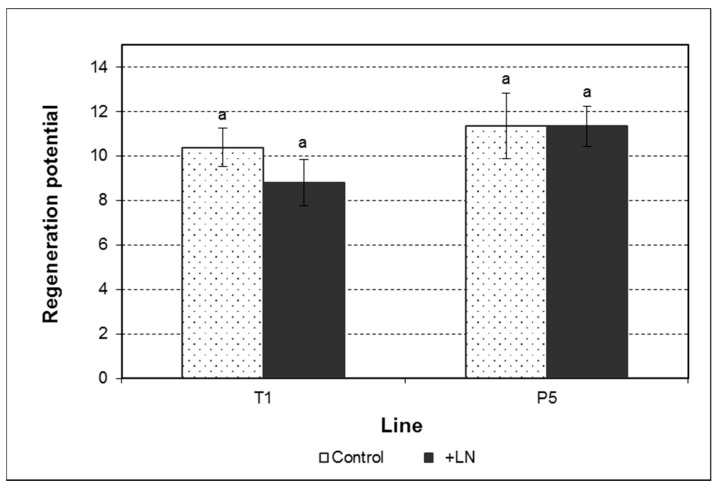
Regeneration potential of control and cryopreserved embryogenic cultures of the lines T1 and P5. Data represent the mean ± SEM. Different letters indicate significant differences by the least significant difference (LSD) test with a significance level of 0.05.

The regeneration potential ranged from 8.80 to 11.35 somatic embryos giving rise to shoots or shoots and roots per culture initiated in the maturation phase (Figure 2). This parameter was not influenced by the genotype, cryopreservation, or the interaction between these two factors (Table S1). Thus, no significant differences were found between the lines tested or between control cultures and those established from cryopreserved somatic embryos. Nevertheless, the genotype had a significant influence on the number of shoots developed per germinated embryo, with 1.72 and 1.71 shoots for control and cryopreserved cultures of the line P5 versus 1.45 and 1.56 shoots for those of the line T1 (Table 1, Table S1). Furthermore, shoots obtained in the line P5 were significantly longer than those developed from somatic embryos of the line T1 (Table 1). While in the line P5 shoots ranged from 11.81 to 12.75 mm, in the line T1, their length averaged 9.27 and 9.17 mm in control and cryopreserved cultures, respectively. No effect of cryopreservation or of the interaction between genotype and cryopreservation could be observed on the quality of the regenerated plants (Table S1).

### 2.2. Effect of Cryopreservation on Ex Vitro Establishment of Regenerated Plants

#### 2.2.1. Shoot Multiplication

**Figure 3:**
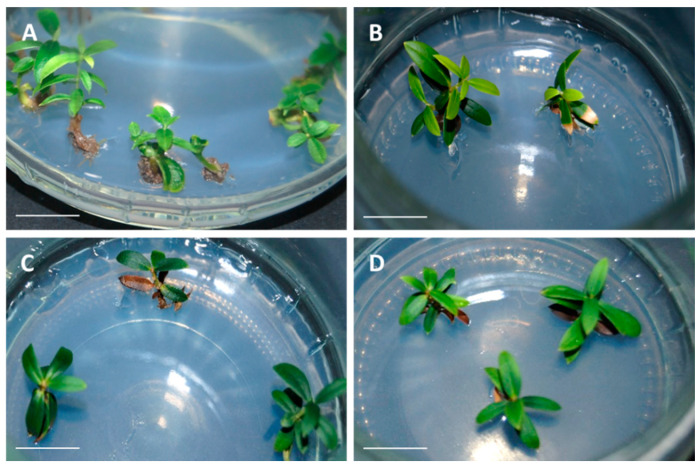
In vitro multiplication of olive plants derived from (**A**,**C**) control and (**B**,**D**) cryopreserved embryogenic cultures of the lines T1 (**A**,**B**) and P5 (**C**,**D**). Bar = 1 cm.

Olive plants showed a good appearance during the multiplication phase, although necrosis sometimes occurred over time in culture, leading to plants loss. No morphological differences due to cryopreservation could be observed in plants derived from each embryogenic line (Figure 3).

According to the statistical analysis, only the number of axillary shoots formed per explant was significantly influenced by the genotype, with more axillary shoots obtained in shoot segments of the line T1 than in those of the line P5 (Table 2; Table S2). Cryopreservation did not influence the multiplication of shoots developed from the germination of olive somatic embryos, not affecting the number of axillary shoots obtained per explant or their length (Table 2; Table S2). However, although no significant differences were observed in the length of the axillary shoots formed, a statistically significant genotype × cryopreservation interaction was found for this variable (Table 2; Table S2). Thus, while in the line T1 cryopreservation provoked a slight increase of the shoot length, from 3.50 to 6.54 mm, in the line P5, a slight decrease could be observed, from 7.56 to 4.81 mm.

#### 2.2.2. Shoot Rooting

**Figure 4:**
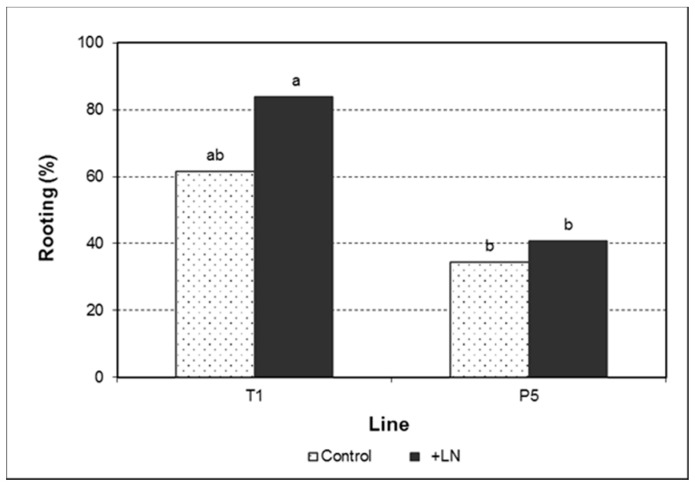
Rooting percentage of olive shoots derived from control and cryopreserved embryogenic cultures of the lines T1 and P5. Different letters indicate significant differences by the R × C test, with a significance level of 0.05.

**Figure 5:**
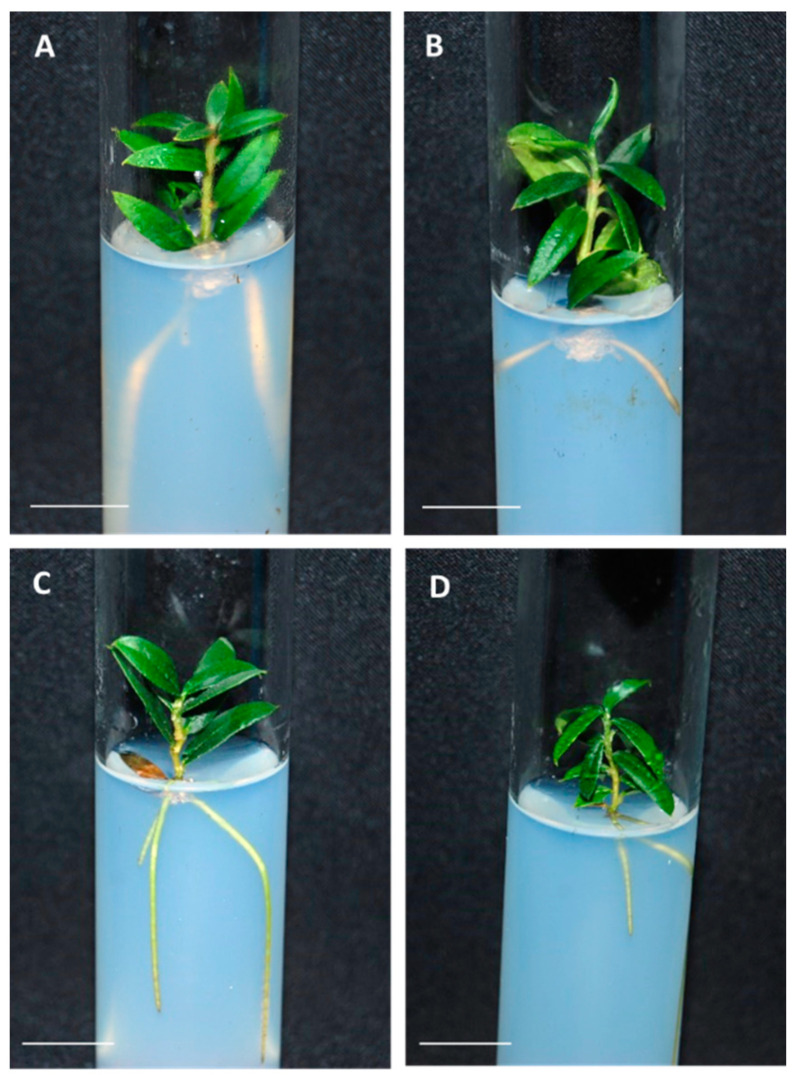
Rooting of olive shoots derived from (**A**,**C**) control and (**B**,**D**) cryopreserved embryogenic cultures of the lines T1 (**A**,**B**) and P5 (**C**,**D**). Bar = 1 cm.

Genotype was the only factor significantly affecting the rooting of the regenerated shoots. Cryopreservation or the genotype × cryopreservation interaction had no influence on this process (Figure 4; Table S2). As revealed by the number of roots developed per shoot segment and root length, rooting quality was not influenced by any predictor variable (Table 3; Table S2). Nevertheless, in both embryogenic lines, cryopreservation was associated with a slight increase in both parameters, giving rise to a higher number of longer roots (Figure 5).

#### 2.2.3. Plant Acclimatization

**Figure 6:**
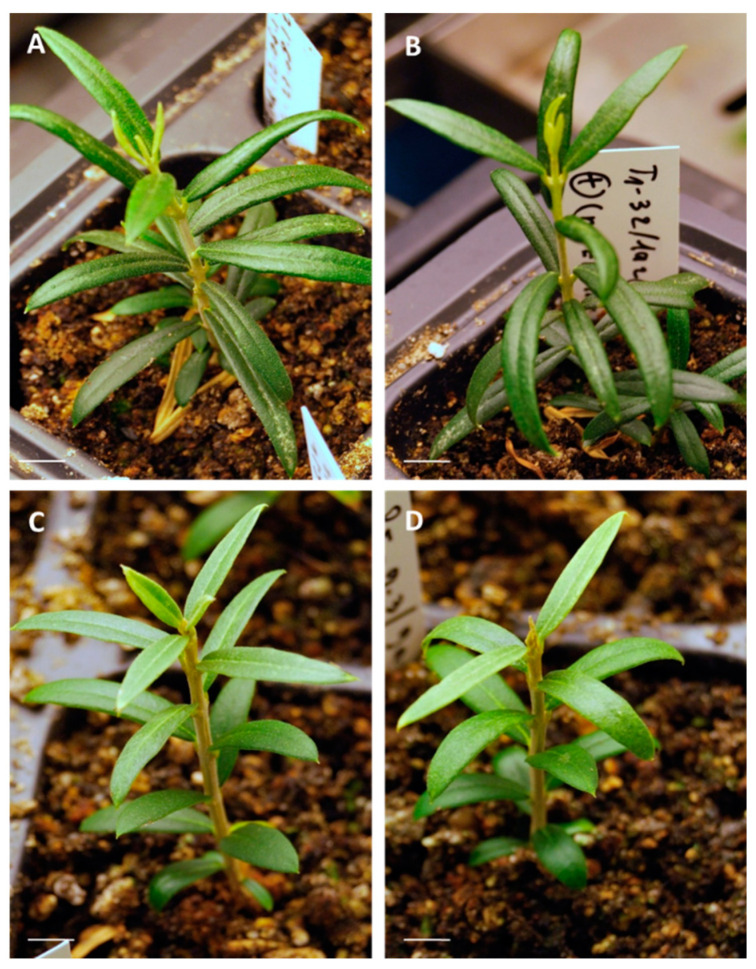
Acclimatization of olive plants developed from (**A**,**C**) control and (**B**,**D**) cryopreserved embryogenic cultures of the lines T1 (**A**,**B**) and P5 (**C**,**D**). Bar = 1 cm.

Independently of their genotype or origin, from control or cryopreserved cultures, somatic plants successfully acclimatized to ex vitro conditions (Figure 6), with acclimatization rates ranging from 93.55 to 100% (data not shown). No significant differences were appreciated due to the genotype, cryopreservation, or the interaction between both factors (Table S2). The acclimatized plants were subsequently transferred to a greenhouse, exhibiting a good appearance and vigorous growth.

## 3. Discussion

Although no effect of the genotype could be observed on the regeneration potential of olive embryogenic cultures, a significant influence on the quality of the regenerated plants was evident. Differences in the vigor of plants regenerated via somatic embryogenesis depending on the genotypic background have been previously reported in olive [19]. The better features observed in plantlets of the line P5 could be explained by differences in the previous maturation phase, which occurred with different efficiencies depending on the embryogenic line [19,20] and largely determines the final output of the somatic embryogenesis process and the quality of the regenerated plants [21].

As previously reported, the genotype also played a primary role in the in vitro steps required for the ex vitro establishment of olive plants regenerated through somatic embryogenesis [19], significantly influencing different aspects of the multiplication and rooting phases. In fact, according to Duong et al. [22], the genotype is one of the factors contributing to the success of micropropagation protocols. However, contrarily to that observed during plant regeneration, better results were obtained in plantlets of the line T1. The shoots derived from embryogenic cultures of this line developed a higher number of axillary shoots per shoot segment during in vitro multiplication and rooted at a higher rate than those regenerated from cultures of the line P5.

Cryopreservation did not influence the regeneration potential of the embryogenic lines tested. The quality of the regenerated plants was not affected either, as no significant differences in the number of shoots per germinated embryos or in shoot length were observed between plants regenerated from control and cryopreserved cultures.

Independent investigations on a broad range of crop species have shown no effect of cryopreservation on the morphogenic potential of embryogenic cultures in terms of plant regeneration capacity [11,23]. Thus, no influence was found in *Vitis* spp. [24], *Gentiana tibetica* [25], *Gentiana cruciata* [25,26], *Coriandrum sativum* [27], *Theobroma cacao* [28], *Pinus radiata* [29], or *Cryptomeria japonica* [30]. Nevertheless, regeneration enhancement has also been described after cryopreservation. This response was observed in embryogenic cultures of different species, such as *Citrus deliciosa* [31], *Festuca arundinacea* [32], *Hevea brasiliensis* [33], *Vitis vinifera* [34], *Cyclamen persicum* [10], *Manihot esculenta* [35], and *G. cruciata* [26]. Interestingly, Lardet [36] showed acquisition of regeneration competence in cell lines of *H. brasiliensis* that initially did not exhibit embryogenic features, thus concluding that cryopreservation improves the morphogenetic competence of embryos derived from a cryopreserved callus. Although less frequent, negative effects of cryopreservation on somatic embryogenesis have also been found. Reduction of morphogenic potential was reported in *Quercus robur* [13] and *Pinus pinaster* [37], and loss of embryogenic potential after cryopreservation was observed in *H. brasiliensis* [36] and *Quercus ilex* [38]. Nevertheless, detrimental effects of cryostorage have been attributed to inappropriate cryopreservation protocols [39] rather than to a negative effect of the cryopreservation process itself. Therefore, optimization of the cryopreservation procedures or of some of the steps included in this conservation technique, such as cryoprotection, may contribute to minimize these negative effects.

Within each embryogenic line, somatic plants developed from control and cryopreserved cultures exhibited the same behavior in the subsequent multiplication, rooting, and acclimatization steps, thus revealing that ex vitro plant establishment was not influenced by cryopreservation. Nevertheless, a significant genotype × cryopreservation interaction was found for shoot length during the multiplication phase. No significant differences were observed in the parameters assessed in the rooting and acclimatization phases. Nonetheless, slightly higher values of rooting percentage, number of roots per rooted shoot, and root length were achieved in shoots derived from cryopreserved cultures of the lines T1 and P5, compared to those obtained in control, non-frozen cultures. Although some works have investigated the influence of cryopreservation on micropropagation and rooting of shoot apices [40], to our knowledge, no reports are available about shoots regenerated via somatic embryogenesis.

In conclusion, cryopreservation did not affect the regeneration potential of olive embryogenic cultures, and the quality of plants regenerated from cryopreserved cultures was similar to that of plants derived from control cultures. The subsequent multiplication, rooting, and acclimatization phases were not affected by cryopreservation either, thus allowing the successful ex vitro establishment of olive plants regenerated via somatic embryogenesis. Therefore, this investigation revealed that the cryopreservation procedure optimized by Bradaï et al. [17] could be efficiently used for the long-term conservation of olive embryogenic lines. Furthermore, the high performance of both protocols, for somatic embryogenesis [41] and cryopreservation [17], and the absence of negative effects due to cryopreservation confirm that the combined use of somatic embryogenesis and cryopreservation can be a reliable option to support olive breeding programs based on both conventional and biotechnological methods.

## 4. Materials and Methods

### 4.1. Plant Material

Olive (*O. europaea* L.) embryogenic cultures were induced from the radicle of mature zygotic embryos, cv. Picual, following the protocol of Orinos and Mitrakos [42]. Embryogenic cultures initiated from single zygotic embryos were maintained as independent lines by repetitive embryogenesis in olive cyclic embryogenesis medium (ECO) [43,44]. The ECO medium consisted of the macronutrients of the OMe medium [45], ¼ MS microelements [46], ½ OM vitamins [47], 50 mg L^−1^ myo-inositol, 58.43 mM sucrose, 550 mg L^−1^ glutamine, and the supplements proposed by Rugini and Caricato [48], i.e., 0.25 μM indole-3-butyric acid (IBA), 0.44 μM 6-benzylaminopurine (BAP), 0.5 μM 2-isopentenyladenine (2 iP), 200 mg L^−1^ cefotaxime, and 1 g L^−1^ casein hydrolysate. Cefotaxime was filter-sterilized and added to the cooled sterilized media. Cultures were incubated at 25 ± 1 °C in darkness. Subcultures were performed at 6–7-week intervals.

The pH of all culture media was adjusted to 5.74 before adding the gelling agent, consisting of agar 6 g L^−1^, except for the ECO medium, which was gelled with phytagel, 3 g L^−1^. Media sterilization was carried out by autoclaving for 20 min at 121 °C and 0.1 MPa.

### 4.2. Somatic Embryo Cryopreservation

Somatic embryo cryopreservation was carried out following the protocol optimized by Bradai et al. [17], using the droplet-vitrification method on aluminum foil strips. One hundred milligram of somatic embryos 1–6 mm in size harvested at the end of a maintenance cycle were incubated in approximately 10 mL of loading solution (LS), consisting of 2 M glycerol and 0.4 M sucrose in basal ECO medium, i.e., without growth regulators and cefotaxime (pH 5.74). After 20 min in darkness at room temperature, LS was replaced by ice-cooled PVS2 solution, consisting of 3.26 M glycerol, 2.42 M ethylene glycol, 1.9 M dimethyl sulfoxide, and 0.4 M sucrose in basal ECO medium (pH 5.74). Somatic embryos dehydrated in PVS2 for 30 min at 0 °C were placed in a droplet of PVS2 solution on an aluminum foil strip and rapidly plunged into LN. After at least 30 min, the samples were rewarmed by incubation in basal ECO medium containing 1.2 M sucrose (pH 5.74) for 15 min at room temperature. Subsequently, they were transferred onto two sterile filter paper discs on top of ECO medium containing 0.3 M sucrose and 0.001% (*w*/*v*) ascorbic acid. One day later, the explants were transferred to standard proliferation medium [19] and incubated at 25 ± 1 °C in darkness.

### 4.3. Effect of Cryopreservation on the Regeneration Potential

In order to evaluate the influence of cryopreservation on the final performance of the somatic embryogenesis process, the regeneration potential was determined in control and cryopreserved cultures of the lines T1 and P5. In accordance with Bradaï et al. [19], the regeneration potential was estimated as the number of somatic embryos per culture initiated in the maturation phase that, at the end of the germination step, gave rise to plantlets with shoots or shoots and roots. For this purpose, 8 to 9 months after rewarming, somatic embryo maturation was induced according to Benzekri and Sánchez-Romero [21]. Briefly, 100 mg of embryogenic tissues selected from proliferation medium were transferred to 90 × 25 mm Petri dishes containing 50 mL of basal ECO medium. After 8 weeks at 25 ± 1 °C in the dark, maturated embryos equal or larger than 3 mm of size were individually cultured in 80 × 85 mm jars containing 50 mL of germination medium [49]. Embryo germination was carried out under a 16 h photoperiod and at a 40 μmol m^−2^ s^−1^ irradiance level, provided by Grolux lamps (Sylvania, Erlangen, Germany), for two recultures of 6 weeks each.

To assess the effect of cryopreservation on the quality of the regenerated plants, the number of shoots developed per germinated embryo and their length were recorded at the end of the germination phase.

### 4.4. Effect of Cryopreservation on the Ex Vitro Establishment of Regenerated Plants

To investigate the effect of cryopreservation on ex vitro plant establishment, the behavior of plants regenerated from cultures established from cryopreserved somatic embryos was compared with that of plants obtained from control, non-frozen cultures during the multiplication, rooting, and acclimatization steps. To examine the effect of the genotype, plants regenerated from control and cryopreserved cultures of the lines T1 and P5 were used in this investigation.

Shoots obtained from somatic embryo germination were multiplied and rooted according to the protocol of Revilla et al. [5]. For shoot multiplication, nodal explants 1.0–1.4 cm-long with two lateral buds were cultured in 95 × 60 mm jars containing 40 mL of Driver and Kuniyuki (DKW) medium [50] with 87.64 mM sucrose and supplemented with 4.4 µM BAP and 0.05 µM IBA. Multiplication was carried out under light conditions, with transference to fresh medium at 6–7-week intervals. The number of axillary shoots and their lengths were recorded in two successive subcultures.

Apical shoots at least 1.5 cm long were rooted in 25 × 150 mm test tubes containing 25 mL of half-strength DKW medium lacking vitamins and amino acids and including 58.43 mM sucrose and 0.5 µM IBA. The cultures were incubated in darkness for one week and later transferred to light conditions. To evaluate the rooting capacity, rooting percentage, number of roots per rooted shoot and length of the roots formed were measured 8 weeks after rooting initiation.

Acclimatization to the ex vitro conditions was carried out as previously indicated [19]. Plants showing a well-developed root system were carefully washed and planted in 5.5 × 5.5 × 10 cm trays containing a mixture of peat and perlite (1:1). The trays were placed into a vessel containing water, which was totally covered with a transparent plastic film and placed into a growth chamber at 25 °C, 60% relative humidity and under a 16 h light photoperiod (65 μmol m^−2^ s^−1^ irradiance). The relative humidity was gradually lowered for 4 weeks. Frequency of survival and plant aspect were recorded 4 weeks later.

### 4.5. Data Recording and Statistical Analysis

For determining the regeneration potential, 20 cultures were initiated in the maturation phase per treatment and embryogenic line, and the experiment was repeated twice. The number of regenerated plantlets subjected to the subsequent multiplication, rooting, and acclimatization steps was variable, depending on the results of the previous phase, very different in the treatments and lines tested. The shoots used per treatment and embryogenic line in the multiplication and rooting phases ranged from 64 to 450 and from 13 to 37, respectively. Eight to 31 plants were acclimated to the ex vitro conditions.

Percentage data were analyzed through frequency analysis with an R × C test of independence or a three-way log-linear analysis, using the BIOMstat software (Exeter Software, Setauket, NY, USA). The rest of the data were subjected to analysis of variance (ANOVA), and differences among treatments were estimated by the LSD test, using the Statistix 10 program (Analytical Software, Tallahassee, FL, USA). The significance level used was 0.05 in all cases [51].

## Figures and Tables

**Table 1:** Effect of cryopreservation on the regeneration quality of control and cryopreserved embryogenic cultures of the lines T1 and P5. Data represent the mean ± SEM. Different letters indicate significant differences by the LSD test, with a significance level of 0.05.

Embryogenic Line	Cryopreservation	Number of Shoots Per Germinated Embryo	Shoot Length (mm)
T1	Control	1.45 ± 0.06 b	9.27 ± 0.46 b
	+ LN	1.56 ± 0.09 ab	9.17 ± 0.46 b
P5	Control	1.72 ± 0.06 a	11.81 ± 0.40 a
	+ LN	1.71 ± 0.05 a	12.75 ± 0.43 a

**Table 2:** Effect of cryopreservation on in vitro multiplication of olive shoots derived from control and cryopreserved embryogenic cultures of the lines T1 and P5. Data represent the mean ± SEM. Different letters indicate significant differences by the LSD test, with a significance level of 0.05.

Embryogenic Line	Cryopreservation	Number of Axillary Shoots Per Explant	Shoot Length (mm)
T1	Control	0.16 ± 0.06 a	3.50 ± 1.04 a
	+LN	0.14 ± 0.04 a	6.54 ± 1.65 a
P5	Control	0.04 ± 0.01 b	7.56 ± 1.44 a
	+LN	0.04 ± 0.01 b	4.81 ± 1.98 a

**Table 3:** Effect of cryopreservation on the rooting capacity of olive shoots derived from control and cryopreserved embryogenic cultures of the lines T1 and P5. Data represent the mean ± SEM. Different letters indicate significant differences by the LSD test, with a significance level of 0.05.

Embryogenic Line	Cryopreservation	Number of Roots Per Rooted Shoot	Root Length (mm)
T1	Control	1.25 ± 0.25 a	30.13 ± 5.61 a
	+LN	2.53 ± 0.27 a	124.32 ± 21.38 a
P5	Control	1.90 ± 0.23 a	105.65 ± 23.22 a
	+LN	2.33 ± 0.80 a	132.77 ± 18.27 a

## Supplementary Materials

The following are available online at https://www.mdpi.com/2223-7747/10/2/396/s1, Table S1: Significance by two-way ANOVA of the single and combined effects of cryopreservation and genotype on the parameters related to the regeneration capacity of olive embryogenic cultures. Table S2: Significance by two-way ANOVA and three-way log-linear analysis of the single and combined effects of cryopreservation and genotype during ex vitro plant establishment.
