# Supplementary material for: Effect of Cryopreservation on the Ex Vitro Establishment of Olive Plants Regenerated via Somatic Embryogenesis
Source: Plants (Basel). 2021 Feb 19;10(2):396. doi: 10.3390/plants10020396 (PMC7921972; doi:10.3390/plants10020396)
Supplement: Supplementary file 1 [file plants-10-00396-s001.pdf]

Table S1. Significance by two-way ANOVA of the single and combined effects of cryopreservation and genotype on the parameters related to the regeneration capacity of olive embryogenic cultures.

**Predictor variable**	**Regeneration potential**	**Number of shoots per germinated embryo**	**Shoot length (mm)**
Line	0.1028	0.0009	0.0000
Cryopreservation	0.4553	0.5550	0.3789
Line x cryopreservation	0.4618	0.4141	0.2799

Table S2. Significance by two-way ANOVA and three-way log-linear analysis of the single and combined effects of cryopreservation and genotype during ex vitro plant establishment.

**Predictor variable**	**Number of axillary shoots per explant**	**Shoot length (mm)**	**Rooting percentage**	**Number of roots per shoot**	**Root length (mm)**	**Acclimatization percentage**
Line	0.0000	0.4060	0.0004	0.6154	0.1844	0.5333
Cryopreservation	0.6850	0.9160	0.2464	0.0595	0.0575	0.6232
Line x cryopreservation	0.6680	0.0430	0.3215	0.3442	0.2874	1.0000
